# Molecular basis for thermal stability and affinity in a VHH: Contribution of the framework region and its influence in the conformation of the CDR3

**DOI:** 10.1002/pro.4450

**Published:** 2022-10-26

**Authors:** Seisho Kinoshita, Makoto Nakakido, Chinatsu Mori, Daisuke Kuroda, Jose M.M. Caaveiro, Kouhei Tsumoto

**Affiliations:** ^1^ Department of Bioengineering, School of Engineering The University of Tokyo Tokyo Japan; ^2^ Department of Chemistry and Biotechnology, School of Engineering The University of Tokyo Tokyo Japan; ^3^ Research Center for Drug and Vaccine Development National Institute of Infectious Diseases Tokyo Japan; ^4^ Laboratory of Global Healthcare, Graduate School of Pharmaceutical Sciences Kyushu University Fukuoka Japan; ^5^ Medical Proteomics Laboratory, The Institute of Medical Science The University of Tokyo Tokyo Japan

**Keywords:** affinity, CDR‐grafting, Nanobody, single domain antibody, thermal stability, VHH, VHH humanization

## Abstract

The camelid single domain antibody, referred to VHH or Nanobody, is considered a versatile tool for various biotechnological and clinical applications because of its favorable biophysical properties. To take advantage of these characteristics and for its application in biotechnology and therapy, research on VHH engineering is currently vigorously conducted. To humanize a camelid VHH, we performed complementarity determining region (CDR) grafting using a humanized VHH currently in clinical trials, and investigated the effects of these changes on the biophysical properties of the resulting VHH. The chimeric VHH exhibited a significant decrease in affinity and thermal stability and a large conformational change in the CDR3. To elucidate the molecular basis for these changes, we performed mutational analyses on the framework regions revealing the contribution of individual residues within the framework region. It is demonstrated that the mutations resulted in the loss of affinity and lower thermal stability, revealing the significance of bulky residues in the vicinity of the CDR3, and the importance of intramolecular interactions between the CDR3 and the framework‐2 region. Subsequently, we performed back‐mutational analyses on the chimeric VHH. Back‐mutations resulted in an increase of the thermal stability and affinity. These data suggested that back‐mutations restored the intramolecular interactions, and proper positioning and/or dynamics of the CDR3, resulting in the gain of thermal stability and affinity. These observations revealed the molecular contribution of the framework region on VHHs and further designability of the framework region of VHHs without modifying the CDRs.

## Abstract 2

PDB Code(s): 7XL0 and 7XL1;

## 1 INTRODUCTION

In the 1990s, functional antibodies devoid of light chains, and composed of only the heavy chain were discovered in the serum of camelids.^1^ The antibodies, referred as heavy‐chain antibodies (HCAbs), are unique because of the absence of the entire light chain and the first heavy chain constant region (CH1). While HCAbs have a molecular mass of 95 kDa, its variable antigen‐binding domain, referred as VHH or Nanobody®, is generally functional as a single domain despite their small size of only 15 kDa.^2^, ^3^

VHHs have typically similar structural characteristics to those of human VH domains, consisting of four framework regions (FR1/2/3/4) surrounding three complementarity determining regions (CDR1/2/3).^3^, ^4^ Compared with conventional murine or human antibodies, VHHs contain CDR3 loops 3–4 residues longer on average, which are also more divergent in both sequence and structure, and occupying a greater range of positions relative to the framework.^5^, ^6^, ^7^, ^8^ In contrast to conventional antibodies, such diversity of CDR3s and their single‐domain nature enable VHHs to form a unique convex‐shape paratope. Since conventional antibodies form a heterodimer (VH‐VL) for their antigen binding domains, the paratope of conventional antibodies is wider and tends to form a flat surface or a groove.^9^ In contrast, convex paratopes of VHHs have smaller antigen‐binding interface and thus VHHs tend to bind to concave‐shaped epitopes, such as enzyme catalytic sites.^6^, ^8^, ^10^, ^11^

VHHs exhibited favorable biochemical characteristics, such as high affinity and specificity (~nM range), high solubility (~10 mg/ml), long shelf life at 4°C (~months), and high expression level in *Escherichia coli*.^2^, ^12^ Some VHHs display an exceptionally stable behavior, resisting temperatures above 90°C.^13^ Based on these characteristics, VHHs are an attractive alternative to antigen‐binding fragments from conventional antibodies such as Fabs and scFvs, in biotechnological, diagnostic, and therapeutic applications.^4^, ^9^, ^14^, ^15^ Indeed, several VHH‐based antibody‐drugs have been developed in clinical trials for human therapy recently.^16^

To be employed in human therapy, VHHs from camelid origin should be subjected to humanization processes to minimize their potential immunogenicity, similarly to other conventional antibodies employed in therapeutics. In a previous study, Cecil Vinkle et al. proposed two general strategies to humanize a VHH: CDR‐Grafting and resurfacing (veneering).^17^ In the CDR‐Grafting strategy, all three CDRs from the camelid VHHs of interest are grafted onto another humanized framework in which non‐human residues are replaced.^17^, ^18^ In the veneering approach, residue substitutions are introduced in the framework regions of the original camelid VHHs of interest, thus mimicking the sequences of human VHs.^17^, ^19^, ^20^

The VHH framework segments exhibit high sequence homology to human VH sequences.^7^, ^21^ The most remarkable difference between VHHs and human VH is the presence of residue substitutions at four positions within framework‐2 (position 42, 49, 50, and 52; IMGT numbering) that are conserved in conventional VH domains and that are involved in forming the hydrophobic interface with VL domains. These four residues have been referred as VHH hallmark residues, and VHHs conserve Phe42, Glu/Gln49, Arg50, and Gly/Leu/Phe52 whereas human VH domains conserve Val42, Gly49, Leu50, and Trp52.^8^, ^19^ A previous study has revealed that humanizing the residues at positions 42 and 52 has a significant effect on the affinity, while humanizing the residues at positions 49 and 50 does not affect dramatically.^17^

In this study, we investigated the impact of CDR‐Grafting using a humanized VHH of therapeutic interest on the affinity for the antigen, its thermal stability, and its structures. Herein we revealed the molecular mechanisms explaining the functional loss of the chimeric VHH upon CDR‐grafting. Further, we analyzed the effect of mutations in framework residues, showing the importance of intramolecular interactions between the CDR3 and framework‐2. In light of these observations, we discuss the contribution of framework on the functionality of VHHs from a molecular perspective, and suggest pathways to engineer humanized VHHs with desired properties.

## 2 RESULTS

### 2.1 VHH humanization through CDR‐Grafting

We selected a humanized VHH, Vobarilizumab, as a model VHH containing a humanized VHH framework. Vobarilizumab, binding interleukin‐6 receptor (IL‐6R), was developed by Ablynx for the treatment of autoimmune diseases and confirmed its low immunogenicity through clinical trials.^22^ As a model VHH for the CDR donor, we selected the llama VHH, 7D12, recognizing epidermal growth factor receptor (EGFR). 7D12 has been isolated from an immune phage library generated by immunization of *Lama glama*.^23^ In a multivalent format, 7D12 blocks ligand‐induced EGFR activation and cellular proliferation,^24^ and the inhibition mechanism has been described at the molecular level by crystal structural analysis.^25^

**FIGURE 1:**
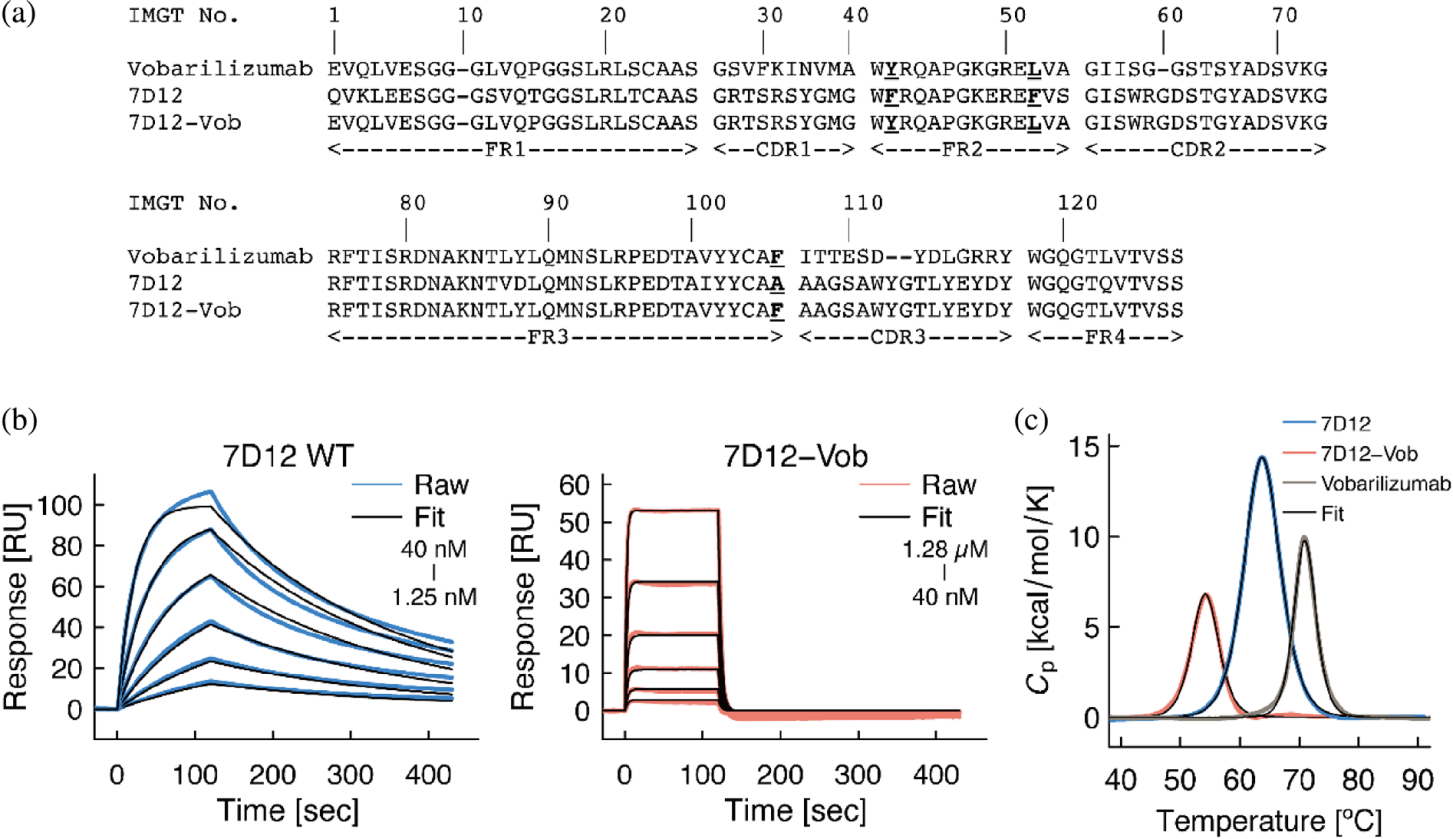
Effect of CDR‐Grafting. (a) Amino acid sequence of wild type VHHs and chimeric VHH 7D12‐Vob. Framework residues at position 42, 52, and 106 are shown in bold and underlined text. CDRs and frameworks are defined according to Saerens, Dirk et al.^31^ (b) Kinetics analysis of the interaction between VHHs and EGFR (cognate antigen of 7D12) by SPR. Raw sensorgram of 7D12 WT, 7D12‐Vob, and fitted sensorgrams are shown in blue, orange, and black lines, respectively. (c) Thermal stability of the VHHs determined by DSC. Representative results are shown

To assess the effects of CDR‐Grafting, we constructed a chimeric VHH, referred to as 7D12‐Vob, by CDR‐grafting from 7D12 onto the framework regions of Vobarilizumab (Figure 1a). We first determined the affinity of wild‐type 7D12 and 7D12‐Vob for the extracellular domain of EGFR using surface plasmon resonance (SPR). The SPR analysis showed eight‐fold lower association rate constant and 50‐fold higher dissociation rate constant, resulting in a 350‐fold worse dissociation constant, for 7D12‐Vob compared with wild‐type 7D12 (Figure 1b and Table 1). We also determined the thermal stability of the VHHs by differential scanning calorimetry (DSC). The results showed a dramatic decrease in the melting temperature (*T*_m_) of 7D12‐Vob (52.2°C) compared with both, wild‐type 7D12 (63.6°C) and Vobarilizumab (70.9°C) (Figure 1c, Table 1). This significant drop of thermal stability upon CDR grafting indicates little compatibility between the CDR and framework regions. The loss of affinity and thermal stability of 7D12‐Vob demonstrate unfavorable effects of CDR‐Grafting onto framework regions of Vobarilizumab on both functional and physicochemical properties.

**TABLE 1:** Kinetics parameters and melting temperature (*T*_m_) upon CDR‐Grafting^a^

	*k*_on_ [×10^5^ M^−1^ s^−1^]	*k*_off_ [×10^−3^ s^−1^]	*K*_D_ [×10^−9^ M]	*T*_m_ [°C]
7D12 WT	12.8 ± 1.8	5.17 ± 1.31	4.10 ± 1.13	63.6 ± 0.3
7D12‐Vob	1.69 ± 0.06	247 ± 22	1,450 ± 90	52.2 ± 1.5
Vobarilizumab	N/A	N/A	N/A	70.9 ± 0.1

^**a**^ Averages and standard deviations of three independent measurements are shown.

### 2.2 Conformational changes of CDR3 upon CDR‐grafting

**FIGURE 2:**
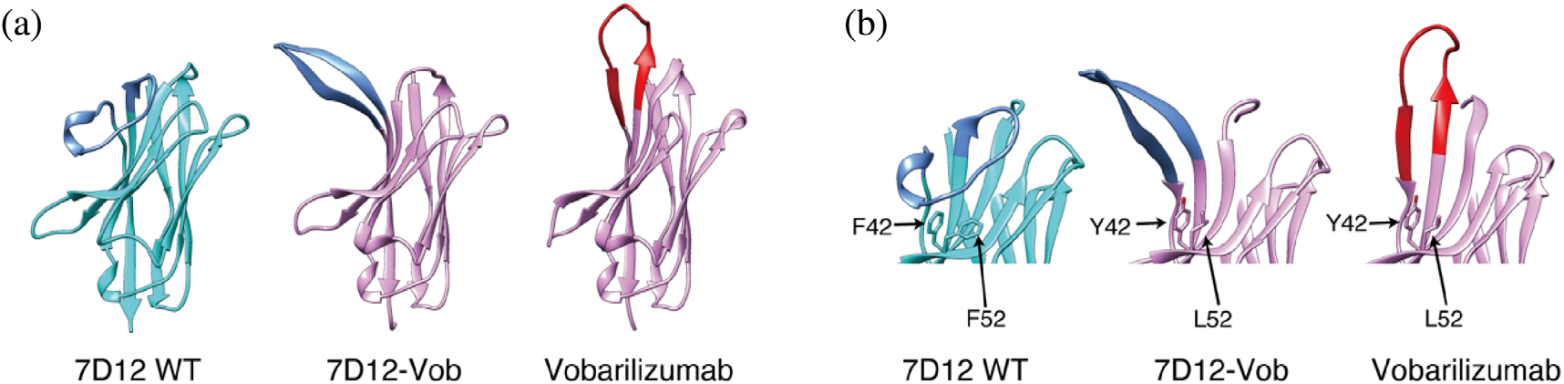
Structures of antibodies. (a) The crystal structure of VHHs, 7D12 WT (PDB ID: 4KRM, bound state), 7D12‐Vob, and Vobarilizumab in the unbound state and (b) close‐up view of CDR. CDR3 of 7D12 and 7D12‐Vob are shown in blue and CDR3 of Vobarilizumab was shown in red. Protein structures were visualized with UCSF Chimera^51^

To gain insight into the molecular basis by which CDR‐Grafting diminished functional and physicochemical properties of the chimeric VHH, we determined the crystal structures of 7D12‐Vob and Vobarilizumab in the unbound state. Although the framework region of 7D12‐Vob exhibited a similar structure to that of wild‐type 7D12 (RMSD Cα: 0.63 Å), the CDR3 loop of 7D12‐Vob adopted an extended conformation different from that of wild‐type 7D12,^25^ which displays a bent CDR3 loop (Figure 2a). Such extended conformation in the CDR3 was also observed in Vobarilizumab, suggesting its contribution of the framework region to the conformation of the CDR3 of the chimeric VHH. Interestingly, whereas framework‐2 is covered by the long CDR3 in wild‐type 7D12, as shown in a previous study,^25^ framework‐2 in the chimeric antibody was exposed due to the extended conformation of its CDR3 (Figure 2b).

We calculated buried surface area (BSA) using PDBePISA^26^ to evaluate the interaction between framework‐2 and CDR3 from the crystal structures. The analysis of BSA residue‐by‐residue indicated that in 7D12, residues Phe42, Arg50, and Phe52 of framework‐2 were involved in the interaction with the CDR3 (BSA values were 45.5 Å^2^, 26.1 Å^2^, and 53.0 Å^2^, respectively). On the other hand, only Tyr42 among these three positions (42, 50, and 52) was involved in contacts with the CDR3 in the crystal structures of 7D12‐Vob and Vobarilizumab, and with lower values (BSA values were 22.9 Å^2^ and 9.6 Å^2^, respectively).

**FIGURE 3:**
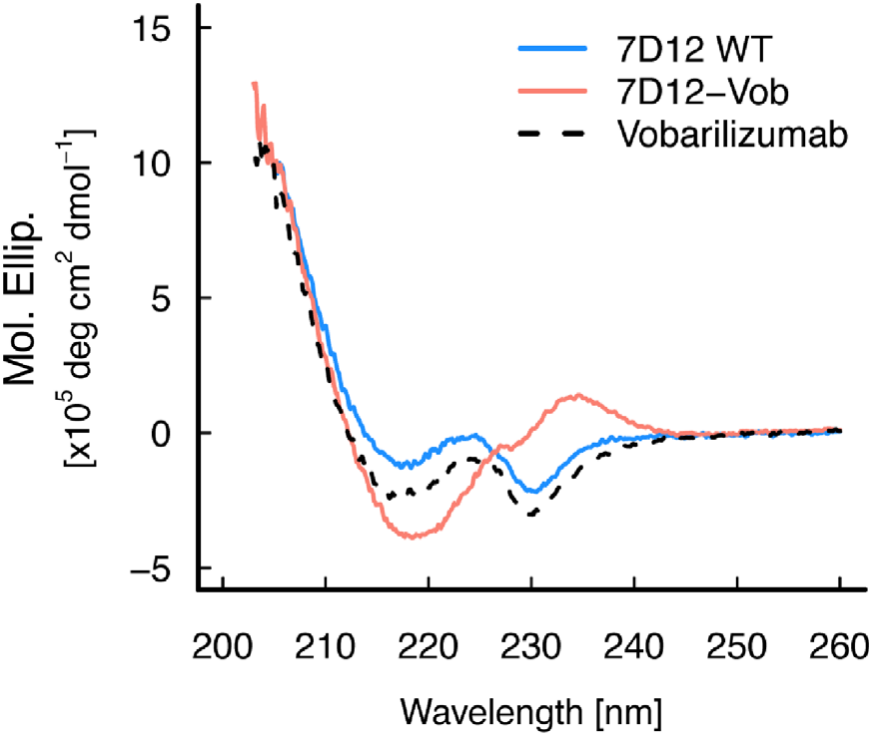
CD spectra of VHHs. Data for wild‐type 7D12, 7D12‐Vob, and Vobarilizumab are shown in blue solid line, red solid line, and black dotted line, respectively

We also collected the circular dichroism (CD) spectra of the VHHs to assess the effects of CDR‐Grafting on the structure of VHH in solution. The results showed that the CD spectrum of 7D12‐Vob exhibited a different profile from that of wild‐type 7D12 or from that of Vobarilizumab (Figure 3). This difference would suggest that conformational changes were induced by CDR grafting, consistent with the differences observed in the crystal structure of 7D12‐Vob.

### 2.3 Affinity and thermal stability loss by substitution of residue 106 at the base of CDR3

**FIGURE 4:**
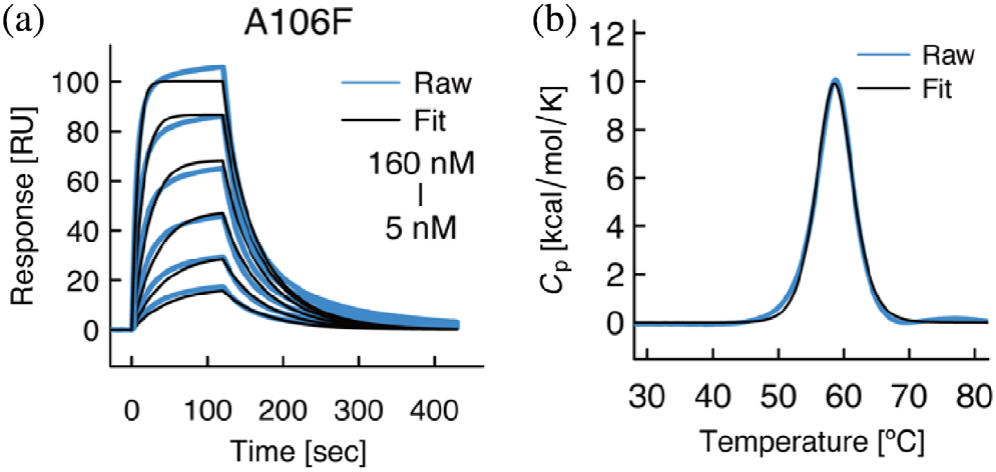
Physicochemical analyses of the 7D12 mutant A106F. (a) SPR and (b) DSC analysis of 7D12 A106F mutant. Raw and fitted sensorgrams are shown with blue and black lines, respectively

We next sought to explain the molecular basis of the conformational change of CDR3 observed in the crystal structures, and for that we employed site‐directed mutagenesis to substitute specific residues of potential interest. We focused on the residue at position 106 (IMGT Numbering) located at the end of framework‐3 and at the base of CDR3 as a possible factor influencing the conformation of CDR3. In wild‐type 7D12 the identity of the residue at position 106 is Ala, whereas in Vobarilizumab it is Phe, a residue with a bulky and hydrophobic sidechain. The crystal structure of wild‐type 7D12 in complex with the cognate antigen EGFR^25^ indicates that Ala106 was not involved in direct interactions with EGFR (BSA: 0.0 Å^2^ as calculated by PDBePISA). We hypothesized that the substitution of a residue with a small sidechain with another residue displaying a bulky sidechain could affect the conformation or dynamics of the CDR loop. To evaluate the effect of the substitution of this position, we prepared the mutant A106F, and analyzed the physicochemical properties of the mutant in the wild‐type 7D12 antibody. The results showed that the A106F substitution resulted in a loss of affinity (~fivefold) and lower thermal stability (~5°C; Figure 4, Table 2). These results suggest that a bulkier residue at that specific position is detrimental for the behavior of CDR3 upon antigen‐binding.

**TABLE 2:** Kinetics parameters and melting temperature (*T*_m_) upon mutation of 7D12^a^

	*k*_on_ [×10^5^ M^−1^ s^−1^]	*k*_off_ [×10^−2^ s^−1^]	*K*_D_ [×10^−9^ M]	*T*_m_ [°C]
A106F	7.67 ± 2.03	2.14 ± 0.76	27.2 ± 3.8	58.5 ± 0.3
F42Y	12.3 ± 3.1	1.02 ± 0.32	8.27 ± 1.47	54.1 ± 0.2
F52L	1.72 ± 0.62	1.44 ± 0.28	8.97 ± 1.59	52.0 ± 0.2
F42Y‐F52L	46.6 ± 1.2	2.99 ± 0.05	64.0 ± 0.7	42.4 ± 0.5

^**a**^ Averages and standard deviations of three independent measurements are shown.

### 2.4 Intramolecular interactions between CDR3 and residues 42 and 52 in wild‐type 7D12

We sought to further elucidate the factors affecting the conformation of CDR3, focusing on a structural feature in the VHH long CDR3. As mentioned above, the long CDR3 in VHHs often bends, making contacts with residues of framework‐2, and shielding the hydrophobic surface that corresponds to the interaction region of the VL domain in conventional VH‐VL antibodies.^27^ As such, wild‐type 7D12 displays a long CDR3 with a bended conformation, as shown in the crystal structure (Figure 2a).

**FIGURE 5:**
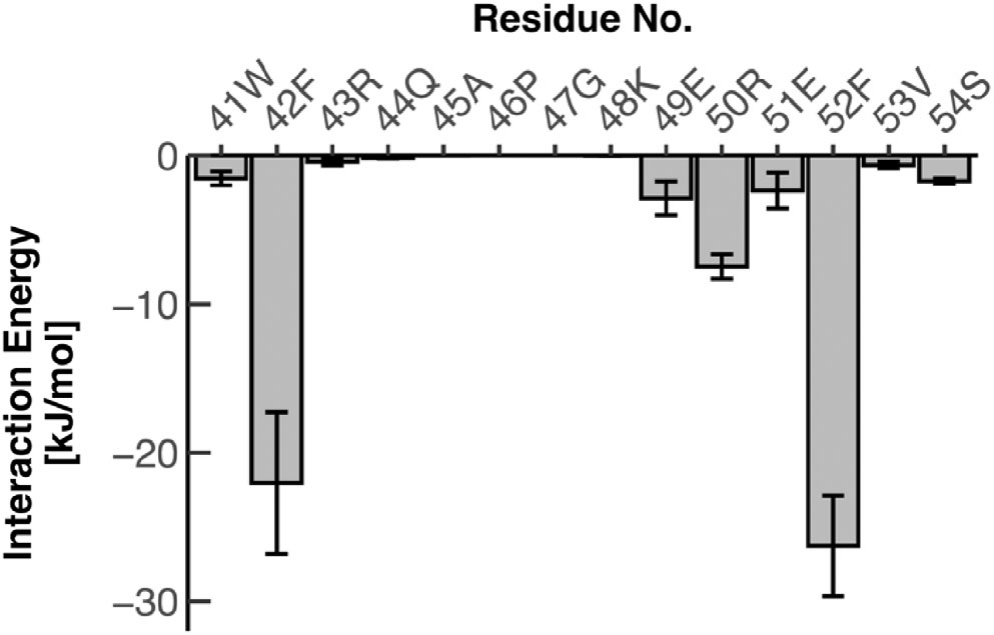
Interaction energy between CDR3 and each residue of framework‐2 calculated from MD simulations. Average and standard deviation values from three independent simulations are shown for each residue. The MD simulations were performed using wild‐type 7D12 without EGFR

To quantitatively evaluate the contribution of the contact region between CDR3 and framework‐2 at the atomic level, we performed molecular dynamics (MD) simulations using the structure of wild‐type 7D12. The interaction energies, namely Lennard‐Jones potential and Coulomb potential, between CDR3 and each residue on framework‐2, were calculated using the gmx_energy module. Large interaction energies were observed for residues 42 and 52, among residues at the framework‐2 (Figure 5), suggesting that these two residues mainly take on the intramolecular interaction between CDR3 and framework‐2.

### 2.5 Importance of Phe42 and Phe52 on physicochemical properties of VHH

**FIGURE 6:**
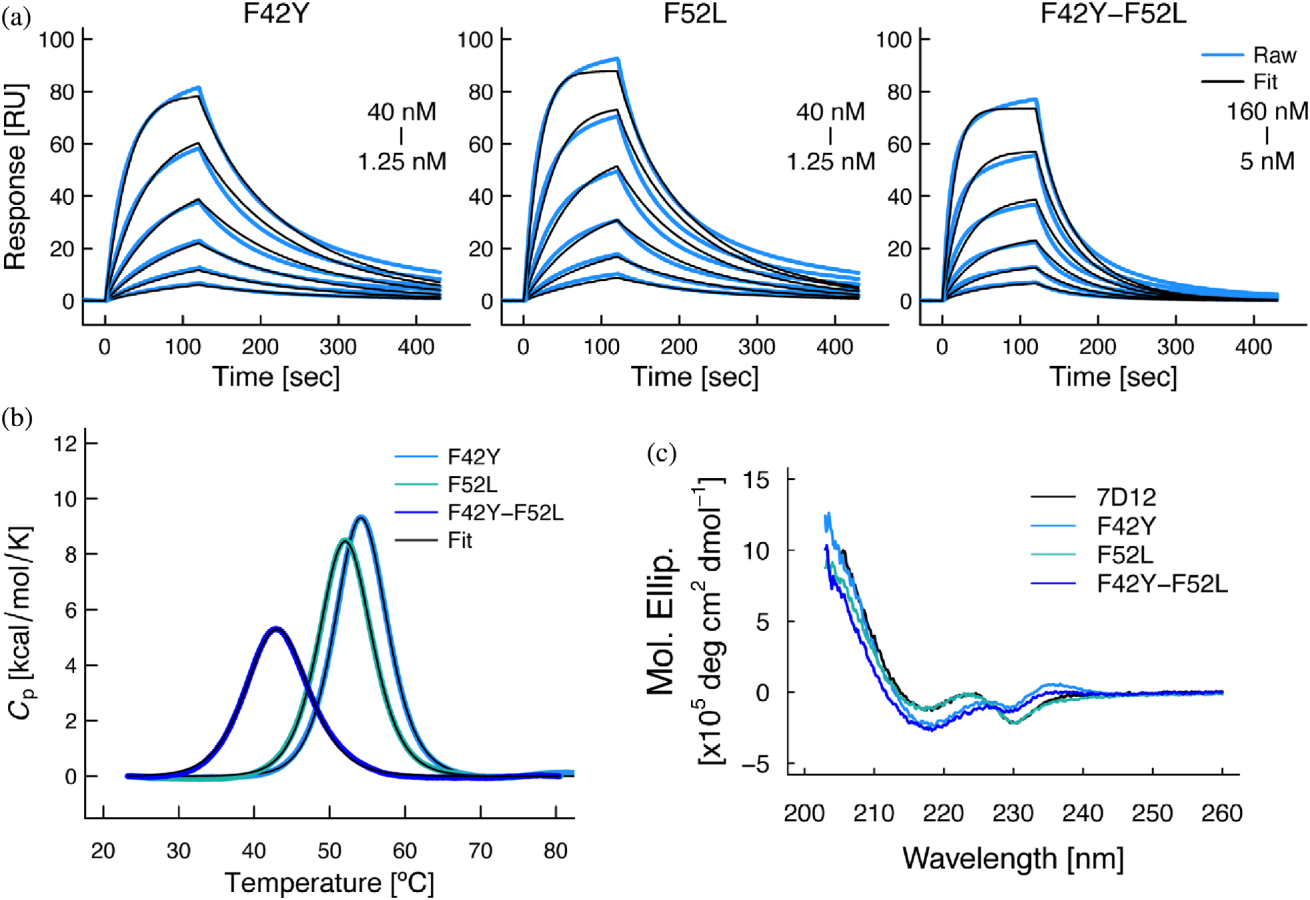
Physicochemical analysis of 7D12 mutants. (a) Binding of 7D12 mutants to immobilized extracellular domain of EGFR evaluated by SPR. (b) Thermal stability of 7D12 mutants as determined by DSC. (c) CD spectra of 7D12 mutants. The spectrum of 7D12‐Vob is shown in black as a reference. Representative results are shown

To validate the importance of intramolecular interactions involving residues Phe42 and Phe52 in wild‐type 7D12, as observed in the MD simulations, mutations were experimentally introduced into wild‐type 7D12 at positions 42 and 52 with the corresponding residues present in Vobarilizumab. Namely, we generated two 7D12 single mutants (F42Y and F52L) and also the double mutant (F42Y‐F52L), and carried out the same biophysical analysis outlined above (determination of affinity with the antigen and thermal stability of the unbound form by SPR and DSC, respectively). Introduction of a single mutation in wild‐type 7D12 led to a two‐fold affinity loss with the antigen (Figure 6a, Table 2), whereas the double mutation led to a 15‐fold affinity loss. In addition, a dramatic decrease of thermal stability was observed in both each of the single mutants (~10°C), and even more in the double mutant (~20°C) (Figure 6b, Table 2). We also obtained the CD spectra of the mutants to evaluate the effects of each mutation on the secondary structures of the VHHs. Compared with wild‐type 7D12, F42Y and F42Y‐F52L mutants exhibited different CD profiles (Figure 6c), suggesting that the mutations induced conformational changes in the VHH. These results are consistent with the observations made in the MD simulations, showing a large contribution of these two residues to maintain the intramolecular interactions between the CDR3 and framework‐2. Collectively, these results suggested that perturbation of the intramolecular interaction network resulted in a significant loss of affinity and even more of the thermal stability of the antibody.

### 2.6 Rescue of affinity and thermal stability upon back‐mutation

As observed above in the antibody 7D12, CDR grafting often results in partial or complete loss of affinity of chimeric antibodies. In such cases, back‐mutations, in which residues of the chimeric antibody are replaced with the corresponding residues in the parental antibody, have been attempted to restore the affinity and stability lost.^28^, ^29^ To apply this strategy to our chimeric VHH (7D12‐Vob), we generated several mutants at the critical positions identified in our analysis above. Thus, Tyr42, and Leu52, and Phe106 of 7D12‐Vob were mutated to Phe42, Phe52, and Ala106, respectively.

**FIGURE 7:**
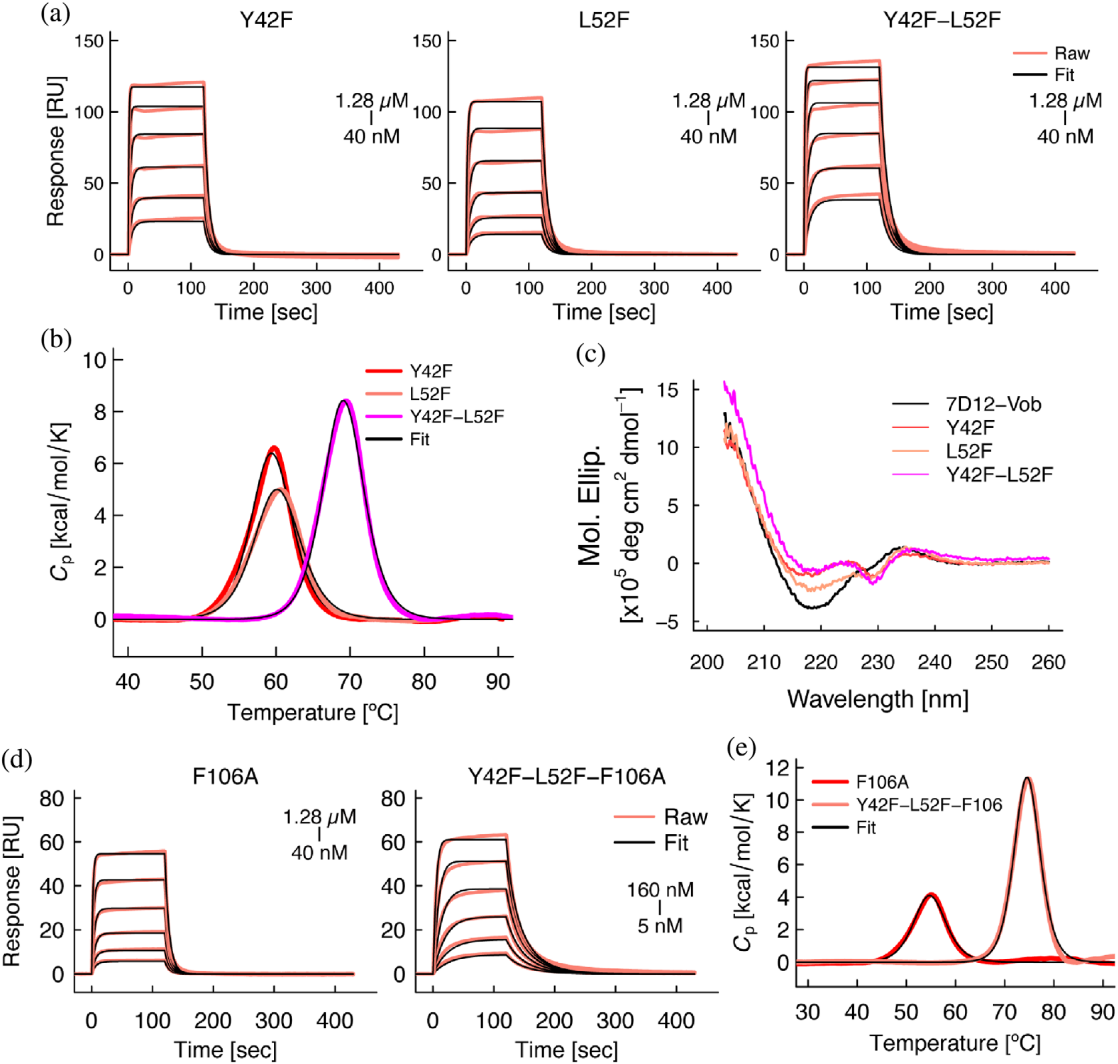
Physicochemical analysis of 7D12‐Vob mutants. (a) Binding of 7D12‐Vob mutants to immobilized extracellular domain of EGFR evaluated by SPR. (b) Thermal stability of 7D12‐Vob mutants as determined by DSC. (c) CD spectra of 7D12‐Vob mutants. The spectrum of 7D12‐Vob is shown in black as a reference. Representative results are shown. (d) Binding of 7D12‐Vob mutants with F106A mutation to immobilized the extracellular domain of EGFR evaluated by SPR. (e) Thermal stability of 7D12‐Vob mutants bearing the F106A mutation as determined by DSC

We examined whether the mutations at residues 42 and 52 led to a reconstitution of the intramolecular interactions between CDR3 and framework‐2, resulting in the restoration of the affinity and/or thermal stability lost upon CDR grafting. SPR and DSC analyses revealed improvements of affinity and thermal stability of the back‐mutants compared with the grafted antibody 7D12‐Vob (Table 3). Higher affinity of the mutants Y42F and Y42F‐L52F mutants (*K*_D_: 191 and 110 nM, respectively) than L52F mutant (*K*_D_: 358 nM) suggested larger effects of the mutation at position 42 on affinity (Figure 7a). In the stability analysis by DSC, the double mutant Y42F‐L52F exhibited higher thermal stability than that of the single mutants, Y42F and L52F (*T*_m_: 69.1°C, 59.5°C, and 60.3°C, respectively), suggesting that residues at position 42 and 52 cooperatively stabilize the chimeric VHH (Figure 7b). The three mutants exhibited different CD profiles compared with 7D12‐Vob (Figure 7c). Intriguingly, Y42F and Y42F‐L52F mutants exhibited CD spectra that were similar to that of the parental wild‐type 7D12 (Figure 3), suggesting that the back‐mutation induced a conformational change in the chimeric antibody that restored the characteristic secondary structure of the parental antibody. Collectively, these results further validate the importance of intramolecular interaction between CDR3 and framework‐2.

**TABLE 3:** Kinetics parameters and melting temperature (*T*_m_) upon mutation of 7D12‐Vob^a^

	*k*_on_ [×10^5^ M^−1^ s^−1^]	*k*_off_ [×10^−2^ s^−1^]	*K*_D_ [×10^−7^ M]	*T*_m_ [°C]
Y42F	7.13 ± 0.21	13.7 ± 0.8	1.91 ± 0.08	59.5 ± 0.7
L52F	3.44 ± 0.54	12.3 ± 1.6	3.58 ± 0.21	60.3 ± 0.1
F106A	3.16 ± 0.24	15.2 ± 0.9	4.82 ± 0.09	55.0 ± 0.6
Y42F‐L52F	7.29 ± 0.39	7.99 ± 0.44	1.10 ± 0.07	69.1 ± 0.8
Y42F‐L52F‐F106A	18.2 ± 0.2	6.93 ± 0.10	0.38 ± 0.01	74.0 ± 0.4

^**a**^ Averages and standard deviations of three independent measurements are shown.

Subsequently, we generated two mutants, 7D12‐Vob F106A and 7D12‐Vob Y42F‐L52F‐F106A, introducing a back‐mutation at the base of CDR3. From SPR and DSC analysis, the F106A mutation resulted in an increase in affinity (~threefold) and thermal stability (3–5°C) (Table 3, Figure 7d,e). As we identified above, the results suggested that the substitution of the bulkier residue at the specific position is detrimental for the behavior of the CDR3.

Although partial recovery of affinity was observed in the individual mutants, the triple mutant 7D12‐Vob Y42F‐L52F‐F106A performed below expectations, exhibiting a 10‐fold lower affinity than the wild‐type 7D12. As additional potential factors explaining the poorer performance, we found that N‐terminal Gln formed a hydrogen bond with the EGFR extracellular domains from the co‐crystal structure of the wild‐type 7D12 and EGFR extracellular domains.^25^ To validate the importance of the hydrogen bond, we prepared the single 7D12‐Vob E1Q mutant and the quadruple 7D12‐Vob E1Q‐Y42F‐L52F‐F106A mutant, and analyzed their physicochemical properties. While E1Q mutation resulted in a slight loss of thermal stability, E1Q led to an increase in affinity (Figure S2, Table S1) indicating the important role upon antigen binding. Indeed, 7D12‐Vob E1Q‐Y42F‐L52F‐F106A exhibited a similar affinity to wild‐type 7D12 (*K*_D_: 4.7 and 4.1 nM respectively), thus recovering the affinity lost upon CDR‐Grafting.

## 3 DISCUSSION

In this study, we assessed the effects of humanization of VHH via CDR‐Grafting. While various humanization campaigns on VHHs have been reported using CDR‐Grafting,^30^ herein we report a new option for CDR‐Grafting using Vobarilizumab, a clinically tested VHH, as a scaffold, and rational VHH engineering. The chimeric VHH 7D12‐Vob, which was generated through CDR‐Grafting, exhibited a notable loss of affinity for the cognate antigen with respect to the parental 7D12 antibody. In addition, the grafted VHH displayed a significant loss of thermal stability with respect to both parental VHHs, 7D12 and Vobarilizumab. The deteriorating biophysical properties of the grafted VHH shed light on the importance of the VHH subfamily and specific residues at positions 42 and 52, in agreement with a previous report.^31^

We investigated the structural aspects of chimeric VHH using crystal structures, MD simulations, and CD spectra to elucidate the molecular basis explaining the suboptimal functionality of the VHH upon CDR‐grafting. Although small conformational changes of CDR loops in antibody humanization have been observed in previous research,^17^, ^19^ the conformational change of the CDR3 loop in 7D12‐Vob was drastic, shifting from a bended conformation, as observed in wild‐type 7D12, to an extended conformation in the grafted VHH. It remained unclear whether this conformational change was facilitated by crystal packing forces, or the absence of its cognate antigen EGFR. However, the significant difference in CD spectra among 7D12‐Vob, 7D12, and Vobarilizumab suggests that the conformational change in 7D12‐Vob also occurs in solution. This structural change is likely a critical factor decreasing both affinity and thermal stability.

The mutation of a residue located at the base of CDR3 (A106F mutation) also resulted in loss of affinity and thermal stability. This mutation indirectly contributes to the interaction, probably by hampering the proper positioning and/or dynamics of the CDR3. As shown in previous studies,^32^, ^33^ the CDR3 itself has a large contribution to the thermal stability of single‐domain antibodies precisely because of the significant proportion of the CDR3 in the overall size of single‐domain antibodies. Further structural analysis of this mutant will provide more detailed molecular insights in the effect of this region of the antibody on the conformation of the CDR3.

Our analysis using MD simulations quantitatively described the contribution of residues at positions 42 and 52 to the intramolecular interactions between CDR3 and framework‐2. Introducing mutations into these positions (F42Y, F52L, or both) resulted in significant loss of affinity and thermal stability, especially in the double mutant (7D12 F42Y‐F52L), suggesting that these two residues, Phe42 and Phe52, cooperatively contributed to its own stability (thermal) and affinity for the antigen. In addition, the CD spectra showed significant effects on the secondary structure of the VHH when mutating residue Phe42 (F42Y mutation). These results suggested the distinct roles of Phe42 and Phe52: Phe42 would play a critical role to maintain the bent conformation of the CDR3, whereas Phe52 would support and stabilize the role of Phe42. While Phe42 would display van der Waals contacts with the atoms in CDR3, our results implied that the mutation F42Y, adding of a hydroxyl group, could disturb these van der Waals contacts.

In the case of VHHs with long CDR3 folding toward the framework, disulfide bond formation between CDR3 and framework‐2, or a hydrogen bond formation between the CDR3 residue and Arg50 at framework‐2 was also observed.^34^ These observations indicate the importance of CDR3 ‐ framework‐2 interaction in VHHs to maintain functionality and superior biophysical properties. In the case of 7D12, Phe42 and Phe52 contributed to the stabilization of the conformation of CDR3. It is noteworthy that Cecil Vinkle et al. had suggested that the humanization of residues at position 42 and 52 had resulted in loss of affinity and/or thermal stability.^17^ Our results revealed the molecular basis explaining the necessity of Phe42 and Phe52, and provided critical insights into the strategy of humanization of VHH antibodies.

In addition to the intramolecular interactions, we also described two additional factors resulting in poorer performance of 7D12‐Vob compared to the parental VHH. As we discussed above, the back‐mutation at the base of CDR3 (F106A mutation) increased the affinity and thermal stability, suggesting that the mutation modulated the proper positioning and/or dynamics of the CDR3. Moreover, an increase in affinity upon introducing a back‐mutation at N‐terminus (E1Q mutation) indicated that interaction between the VHH and EGFR was mediated not only by CDRs but also by the N‐terminal residue.^6^ Coupling with the back‐mutations in the framework region and at the N‐terminus, we restored the loss of affinity upon CDR‐Grafting. The results indicated that the affinity of the VHH is modulated by the residues mediating the behavior and/or conformation of CDR3 as well as the interaction at the N‐terminus of the VHH.

Finally, we demonstrated that the affinity and thermal stability of the VHH could be improved by introducing mutations in the framework region without modifying the CDRs. These results provide important insights into the optimization strategy of humanized framework of VHHs, and moreover as a general strategy for VHH engineering in which the sequence of CDRs should be preserved to maintain their affinity and specificity. Further studies will expand the designability of VHHs based on our exhaustive study.

## 4 METHODS

### 4.1 Protein expression and purification

The DNA sequence encoding each of the VHHs with a C‐terminal hexa‐histidine tag was cloned into an expression vector, pRA2, for bacterial expression. To generate 7D12‐Vob or introduce mutations, a standard inverse PCR method was performed using primers containing each CDR sequence or each of the mutations. The expression and purification of the recombinant VHHs ware carried out based on a previous report.^35^ Briefly, *Escherichia coli* BL21(DE3) cells (Merck; Darmstadt, Germany) were transformed with the plasmid constructs. The recombinant VHHs were extracted from a periplasmic fraction of *E. coli* using ultrasonication, and purified using Ni‐NTA agarose (QIAGEN; Düsseldorf, Germany) equilibrated with the binding buffer (500 mM NaCl, 50 mM Tris–HCl, pH 8.0, containing 5 mM imidazole). The VHHs were eluted with the binding buffer containing increasing concentrations of imidazole (20–500 mM). The eluted fractions were further purified by size‐exclusion chromatography using a HiLoad 26/600 Superdex 75 pg column (Cytiva; MA, USA) equilibrated with PBS buffer.

The DNA sequence encoding the extracellular domain of EGFR (residues 1–618) with an octa‐histidine tag at the C terminus was subcloned into the pFASTBac1 vector (Invitrogen, MA, USA). The extracellular domain of EGFR was expressed using Sf9 expression system (Thermo Fisher Scientific, MA, USA).^25^, ^36^ The bacmid and the baculovirus were prepared according to the protocol provided by the manufacturer (Thermo Fisher Scientific) and a previous report.^37^ The recombinant EGFR was purified from the culture supernatant by using Ni‐NTA as described in the purification of the recombinant VHHs. The eluted fractions were further purified by size‐exclusion chromatography using a HiLoad 26/600 Superdex 200 pg column (Cytiva) equilibrated with PBS buffer.

The protein concentration was determined by absorbance at 280 nm using the computed extinction coefficient of each protein from their amino acid sequence.

### 4.2 Crystallization, data collection, structure determination, and refinement

Purified Vobarilizumab was concentrated using Amicon Ultra MWCO 10,000 (Merck) to 13.4 mg/ml in PBS. The crystallization was performed in the hanging‐drop vapor‐diffusion method at 20°C. Crystals of Vobarilizumab were obtained by mixing drops of 2 μl of protein with 2 μl of the crystallization buffer and equilibrating against the crystallization buffer composed of 0.2 M lithium sulfate and 20% PEG 3350. Separately, purified 7D12‐Vob was concentrated to 6.5 mg/ml in PBS. The crystallization screening was performed by using an Oryx8 protein crystallization robot (Douglas Instruments; Berkshire, UK) with PEG/Ion and PEG/Ion 2 screening kits (Hampton Research, CA, USA). Protein sample (0.5 μl) was mixed with each crystallization buffer (0.5 μl) and equilibrated against a reservoir of the same buffer in the sitting‐drop vapor‐diffusion method at 20°C. Crystals of 7D12‐Vob appeared in a solution containing 0.2 M sodium malonate (pH 4.0) and 20% PEG 3350. Suitable crystals were incubated in a solution containing mother liquor supplemented with 20% glycerol, and subsequently transferred to liquid nitrogen for storage until data collection.

Diffraction data from single crystals were collected in beamline BL1A at the Photon Factory in Tsukuba (Japan) under cryogenic conditions (100 K). Diffraction images were initially processed with MOSFLM,^38^ followed by merging and scaling the data with SCALA of the CCP4 suite.^39^ The structure of Vobarilizumab was determined by the molecular replacement method using the coordinates of another VHH (PDB entry code 5HVG)^40^ with the program PHASER.^41^ The initial model was thoroughly refined with the program REFMAC5^42^ and manually built with COOT.^43^ For 7D12‐Vob, the structure was determined with PHASER using the coordinates of Vobarilizumab as an initial model, and refined as outlined above. Validation was carried out with PROCHECK.^44^ Data collection and structure refinement statistics are given in Table S2. The final models were deposited to Protein Data Bank as PDBID:

### 4.3 Surface plasmon resonance

The interactions of the VHHs with EGFR were analyzed by SPR using a Biacore 8 K instrument (Cytiva). EGFR was immobilized on a CM5 sensor chip (Cytiva) according to the manufacturer's standard amine coupling protocol at around 1000RU. The VHHs were injected onto the sensor chip at a flow rate of 30 μl/min in two‐fold serial dilutions. The association time was 120 s, and the dissociation time was 300 s. The sensor chip was regenerated at the end of each cycle with two 30 s injections of 10 mM Glycine‐HCl buffer at pH 2.5. The measurements were carried out at 25°C using PBS buffer supplemented with 0.005% Tween20. Data were analyzed using Biacore Insight Evaluation Software (Cytiva).

### 4.4 Differential scanning calorimetry

The thermal stability of the VHHs was measured by DSC using a MicroCal PEAQ‐DSC Automated system (Malvern; Worcestershire, UK). The protein samples (1 mg/ml in PBS buffer) were heated from 20°C to 110°C at a scanning rate of 1.0°C/min. The data were analyzed using MicroCal PEAQ‐DSC software (Malvern).

### 4.5 Circular dichroism spectra

Circular dichroism (CD) spectroscopy measurements were carried out using a JASCO J‐820 spectropolarimeter (JASCO; Tokyo, Japan). For the measurements in the far‐UV region, each of the protein samples (30 μM, in PBS buffer) was placed in a 1‐mm quartz cell and measured five times with a bandwidth of 1 nm.

The molar ellipticity (Mol. Ellip. [deg cm^2^ dmol^−1^])of each VHH was determined from^45^:

$$\mathrm{Mol}.\;\text{Ellip}.=100\times \theta /\left(C\times d\right)$$

where *θ* is the observed ellipticity [deg], *C* is the concentration of the protein [M], and *d* is the pathlength [cm].

### 4.6 Molecular dynamics simulation

Molecular dynamics (MD) simulations of 7D12 VHH (Unbound state) were performed using GROMACS2016.3^46^ with the CHARMM36m force field^47^ as previously described.^48^ We eliminated the structures of the extracellular domain of EGFR or other 7D12 VHHs in the PDB file (ID: 4KRM) and created a monomeric 7D12 structure. With this monomeric structure, solvation was performed with TIP3P water^49^ in a rectangular box such that the minimum distance to the edge of the box was 15 Å under periodic boundary conditions through the CHARMM‐GUI.^50^ The protein charge was neutralized with added Na^+^ or Cl^−^, and additional ions were added to imitate a salt solution of concentration 0.14 M. The system was energy‐minimized with 5,000 steps and equilibrated with the NVT ensemble at 298 K for 500 ps. Further simulations were performed with the NPT ensemble for 500 ns. Three independent production runs were performed with different initial velocities, and the last 100 ns of each run were used for subsequent analysis. The interaction energy and root mean square deviations (RMSDs) were computed with the GROMACS package (Figure S1). Five Cα atoms at the N terminus and C terminus were excluded from the calculation of RMSD of Cα atoms.

### 4.7 Accession numbers

The coordinates and structure factors of 7D12‐Vob and Vobarilizumab have been deposited in the Protein Data Bank with entry codes 7XL1 and 7XL0, respectively.

## AUTHOR CONTRIBUTIONS

**Seisho Kinoshita:** Conceptualization (equal); data curation (lead); investigation (lead); visualization (lead); writing – original draft (lead); writing – review and editing (equal). **Makoto Nakakido:** Conceptualization (lead); funding acquisition (equal); project administration (equal); supervision (lead); writing – original draft (equal); writing – review and editing (equal). **Chinatsu Mori:** Data curation (equal); investigation (equal); writing – review and editing (equal). **Daisuke Kuroda:** Conceptualization (equal); software (equal); supervision (equal); writing – review and editing (equal). **Jose Caaveiro:** Formal analysis (equal); investigation (equal); supervision (equal); writing – review and editing (equal). **Kohei Tsumoto:** Funding acquisition (lead); project administration (lead); supervision (equal); writing – review and editing (equal).

## Supporting information


**Appendix S1** The root mean square deviations (RMSDs) of each molecular dynamics simulation with wild‐type 7D12 were shown in Figure S1. Physicochemical analysis of 7D12‐Vob E1Q mutants is shown in FIGURE S2. Kinetic parameters and melting temperature (Tm) upon mutation of 7D12‐Vob E1Q mutants are given in Table S1. Data collection and structure refinement statistics are given in Table S21i  .

**DATA S1** 7XL0

**DATA S2** 7XL1

## Data Availability

The data that support the findings of this study are openly available in Protein Data Bank at https://www.rcsb.org/, reference numbers are 7XL0 and 7XL1.
