# Supplementary material for: Molecular basis for thermal stability and affinity in a VHH: Contribution of the framework region and its influence in the conformation of the CDR3
Source: Protein Sci. 2022 Oct 26;31(11):e4450. doi: 10.1002/pro.4450 (PMC9601775; doi:10.1002/pro.4450)
Supplement: Supplementary file 1 — Appendix S1 The root mean square deviations (RMSDs) of each molecular dynamics simulation with wild‐type 7D12 were shown in Figure S1. Physicochemical analysis of 7D12‐Vob E1Q mutants is shown in FIGURE S2. Kinetic parameters and melting temperature (Tm) upon mutation of 7D12‐Vob E1Q mutants are given in Table S1. Data collection and structure refinement statistics are given in Table S21i . [file PRO-31-e4450-s002.docx]

## Supporting information


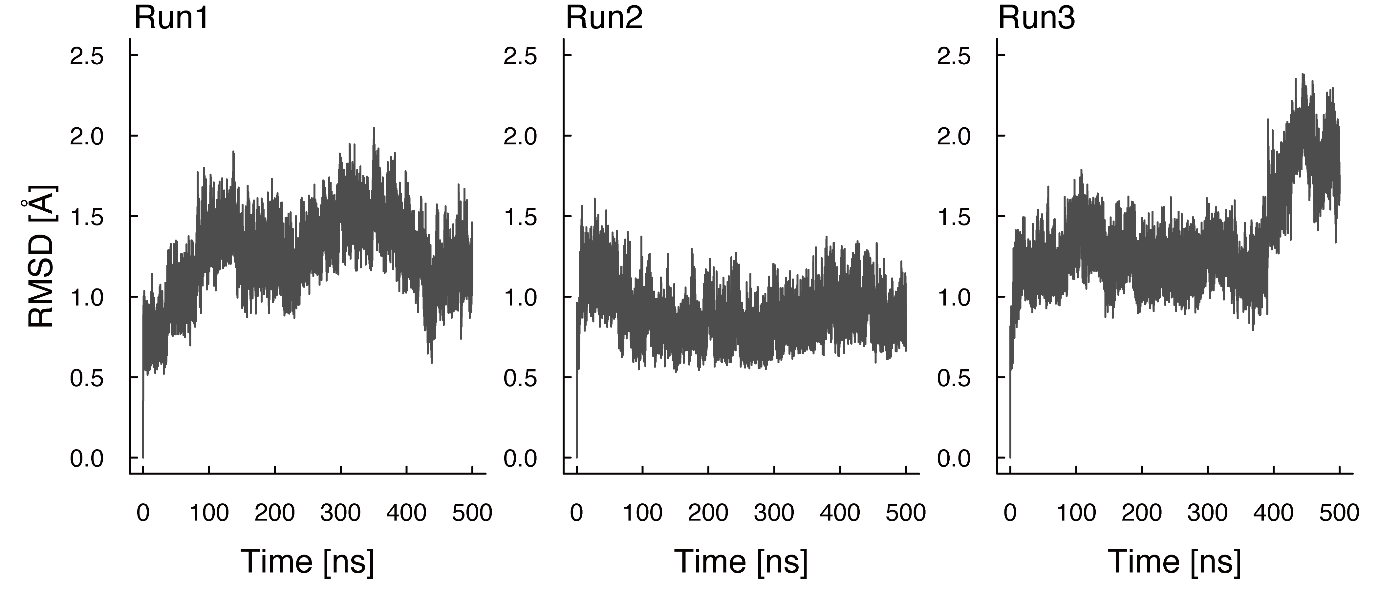


Figure S1 RMSD values of Cα atoms of wild-type 7D12. MD simulations were performed using the co-crystal structure without the cognate antigen, the extracellular domain of EGFR.


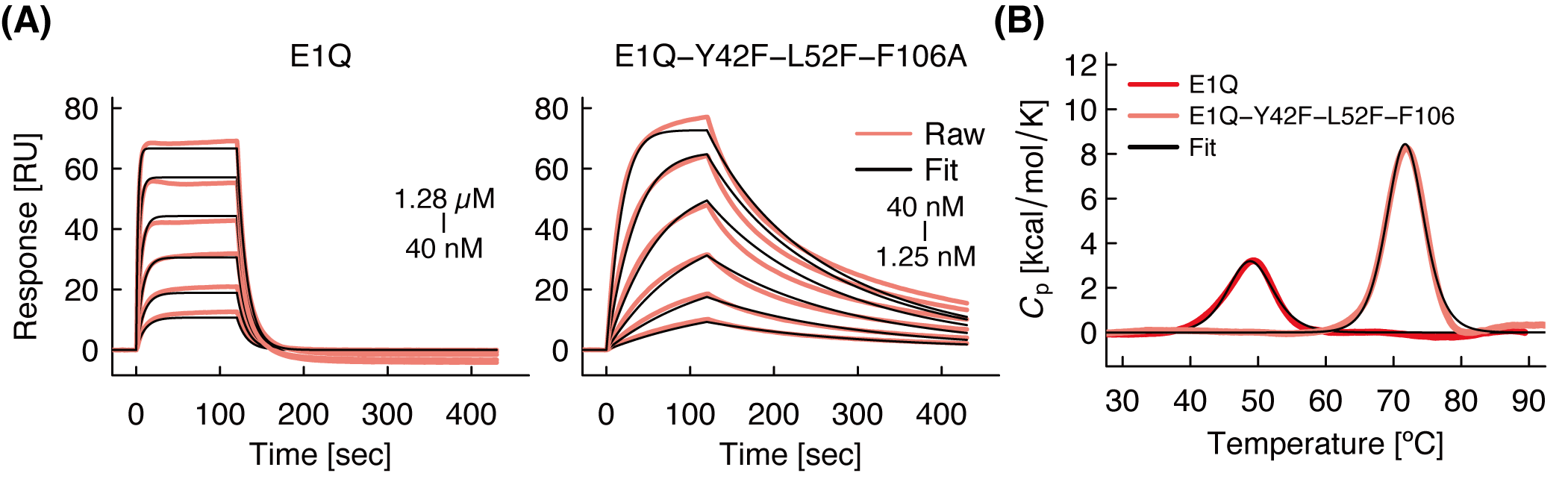


Figure S2 Physicochemical analysis of 7D12-Vob E1Q mutants. (A) Binding of 7D12-Vob mutants bearing the E1Q mutation to immobilized extracellular domain of EGFR evaluated by SPR. (B) Thermal stability of 7D12-Vob mutants with E1Q mutation as determined by DSC.

Table S1 Data collection and refinement statistics.

Statistical values given in parenthesis refer to the highest resolution bin.

| **Data Collection** | **Vobarilizumab** | **7D12-Vob** |
| --- | --- | --- |
| Space Group | C 2 | P 4_2_ 2_1_ 2_1_ |
| Unit cell |  |  |
| a, b, c (Å) | 124.6, 56.4, 42.2 | 68.6, 68.6, 103.7 |
| α, β, γ (°) | 90.0, 101.9, 90.0 | 90.0, 90.0, 90.0 |
| Resolution (Å) | 51.2 – 1.70 | 35.4 - 1.65 |
| Wavelength | 1.1000 | 1.1000 |
| Observations | 187,302 (21,627)) | 261,357 (39,040) |
| Unique reflections | 31,315 (4,232) | 30,042 (4,233) |
| *R_merge._* | 0.092 (0.336) | 0.108 (0.963) |
| *R_p.i.m._* | 0.040 (0.160) | 0.038 (0.327) |
| CC_1/2_ | 0.995 (0.890) | 0.997 (0.724) |
| *I / σ (I)* | 11.5 (4.4) | 12.0 (2.3) |
| Multiplicity | 6.0 (5.1) | 8.7 (9.2) |
| Completeness (%) | 98.9 (92.5) | 98.8 (97.8) |
| **Refinement Statistics** |  |  |
| Resolution (Å) | 51.2 – 1.70 | 35.4 - 1.65 |
| *R_work_* / *R_free_* (%) | 15.2 / 19.6 | 15.6 / 20.6 |
| No. protein chains | 2 | 2 |
| No. atoms |  |  |
| Protein | 1,861 | 1,956 |
| Other (no solvent) | 16 | 14 |
| Water | 188 | 156 |
| B-factor (Å^2^) |  |  |
| Protein | 27.6 | 27.7 |
| Other (no solvent) | 33.7 | 29.8 |
| Water | 34.6 | 34.9 |
| Ramachandran Plot |  |  |
| Preferred (%) | 90.5 | 93.0 |
| Allowed (%) | 9.5 | 7.0 |
| Outliers (%) | 0 | 0 |
| RMSD Bond (Å) | 0.006 | 0.007 |
| RMSD Angle (°) | 1.35 | 1.43 |
| PDB | 7XL0 | 7XL1 |

Table S2 Kinetic parameters and melting temperature (*T*_m_) upon mutation of 7D12-Vob E1Q mutants^a^.

|  | ***k*_on_ [ × 10^5^ M^–1^ s^–1^ ]** | ***k*_off_ [ × 10^–2^ s^–1^ ]** | ***K*_D_ [ × 10^–9^ M ]** | ***T*_m_ [ °C ]** |
| --- | --- | --- | --- | --- |
| **E1Q** | 3.48 ± 0.23 | 8.56 ± 0.14 | 247 ± 12 | 49.4 ± 0.6 |
| **E1Q-Y42F-L52F-F106A** | 18.9 ± 0.8 | 0.89 ± 0.03 | 4.71 ± 0.04 | 72.0 ± 0.4 |

**^a^** Averages and standard deviations of three independent measurements are shown.
